# Does environmental confounding mask pleiotropic effects of a multiple sclerosis susceptibility variant on vitamin D in psychosis?

**DOI:** 10.1038/npjschz.2015.36

**Published:** 2015-10-28

**Authors:** Conrad O Iyegbe, Anita Acharya, John Lally, Poonam Gardner-Sood, Louise S Smith, Shubulade Smith, Robin Murray, Oliver Howes, Fiona Gaughran

**Affiliations:** 1 Department of Psychosis Studies, Institute of Psychiatry, King's College London, London, UK; 2 Barts and The London School of Medicine and Dentistry, Queen Mary University, London, UK; 3 National Psychosis Service, South London and Maudsley NHS Foundation Trust, London, UK; 4 King’s College London Dental Institute, Tower Wing, Guys Hospital, London, UK; 5 Department of Forensic and Neurodevelopmental Science, Institute of Psychiatry, King's College London; 6 Biomedical Research Centre, BRC Nucleus, Maudsley Hospital, South London and Maudsley NHS, London, UK

## Abstract

**Background:**

This work addresses the existing and emerging evidence of overlap within the environmental and genetic profiles of multiple sclerosis (MS) and schizophrenia.

**Aims:**

To investigate whether a genetic risk factor for MS (rs703842), whose variation is indicative of vitamin D status in the disorder, could also be a determinant of vitamin D status in chronic psychosis patients.

**Methods:**

A cohort of 224 chronic psychosis cases was phenotyped and biologically profiled. The relationship between rs703842 and physiological vitamin D status in the blood plasma was assessed by logistic regression. Deficiency was defined as a blood plasma concentration below 10 ng/µl. Potential environmental confounders of the vitamin D status were considered as part of the analysis.

**Results:**

We report suggestive evidence of an association with vitamin D status in established psychosis (*ß*_standardized_=0.51, *P*=0.04). The logistic model fit significantly benefited from controlling for body mass index, depression and ethnicity (*χ*^2^=91.7; 2 degrees of freedom (df); *P*=1.2×10^20^).

**Conclusions:**

The results suggest that, in addition to lifestyle changes that accompany the onset of illness, vitamin D dysregulation in psychosis has a genetic component that links into MS. Further, comprehensive studies are needed to evaluate this prospect.

## Introduction

The discovery of genetic risk markers for schizophrenia on a large scale^1^ permits broader questions to be asked about the genetic architecture of the illness.^2^ Accordingly, recent investigative efforts have sought to determine the extent of overlap between the common genetic architecture of schizophrenia and those of other psychiatric and non-psychiatric traits. A recent study found no evidence of a shared genetic etiology between schizophrenia and Type 2 Diabetes.^3^ There is, on the other hand, persuasive evidence of a genetic link with cardiovascular disease.^4^ The implication of this is that, in addition to lifestyle changes that accompany the onset of schizophrenia, the high burden of cardiovascular disease in schizophrenia is partly due to sharing of genetic factors by both traits. Cross-disorder genomic studies recently revealed the existence of genetic links between schizophrenia and multiple sclerosis (MS; OMIM:126200),^5^ a neurodegenerative condition in which the biological competence of the neuronal system is undermined by chronic autoimmunity. Epidemiological evidence enhances the basis for such a link. For example, a previous study of population registry data has demonstrated MS can be a risk factor for schizophrenia and for non-affective psychosis,^6,7^ whereas a family history of MS has been associated with schizophrenia and non-affective psychosis.^7^ The parallels between schizophrenia and MS even extend to the (non-genetic) risk factors that they share. For instance, both disorders show directionally consistent effects of geography, migration and season on risk.^8–14^ Because (i) these risk factors are also proxies for vitamin D status^15,16^ and (ii) low vitamin D status is a common finding in both conditions,^17,18^ the possibility exists that vitamin D is the factor that explains this symmetry. Vitamin D has been proposed as a susceptibility factor for a number of neuropsychiatric disorders. The strength of substantiating evidence is variable between schizophrenia, autism and Alzheimer’s Disease.^19^ It is strongest however in MS, where earlier suspicions of vitamin D involvement have been confirmed by genome-wide association studies (GWAS).^20,21^ Concrete evidence of vitamin D causality remains elusive in the field of schizophrenia genetics, although calcium signaling is now a recurring theme in its GWAS.^1^ Because one of the key roles of vitamin D is to facilitate the absorption and homeostasis of calcium,^22^ it is now also possible to envisage a role of some kind for vitamin D in the pathogenesis of schizophrenia.

To better understand whether vitamin D deficiency in schizophrenia and MS might be biologically linked, this study tests the possibility that deficiency is partly the result of shared genetic influences (in addition to shared lifestyle effects). The focus of the study is a single-nucleotide polymorphism (rs703842), located on the *CYP27B1* gene (chromosome 12). Support for the contribution of the rs703842 locus to MS susceptibility is unequivocal.^21,23,24^ Furthermore, the encoded gene product, 25-hydroxyvitamin D-1 α-hydrolase (or 1α(OH)ase), is responsible for converting the storage form of vitamin D, 25-hydroxyvitamin D (or 25(OH)D), into the biologically active vitamin D compound, 1,25(OH)_2_D3. The integral nature of the encoded enzyme to vitamin D biosynthesis would suggest that the true causal variant presumably tagged by rs703842 will influence vitamin D levels (and MS risk) through polymorphic variation in enzymic activation. But formal experiments that test this theory have not been reported. Current follow-up work has instead yielded insights into the consequences of rs703842 variation on gene expression. Expression profiles have been investigated using both diseased (MS) and non-diseased populations;^25,26^ there is a reassuring level of consistency between them. For instance, knock-on effects on *FAM119B* transcripts have been identified in both studies. Together, these studies suggest that variation at rs703842 is a conduit to molecular targets other than just vitamin D.

This study explores whether variation at rs703842 is associated with vitamin D deficiency in chronic psychosis, in addition to its role in MS. This is performed by modeling the relationship between the rs703842 polymorphism and vitamin D levels in chronic psychosis patients. An association analysis of rs703842 genotypes and case–control status is not within the scope of the study, as controls are not included in the cohort.

Finally, because physiological vitamin D status is also sensitive to a number of extraneous factors,^16^ our methodological approach assumes that controlling for such effects will promote the detection of association signals that would normally be lost to confounding. Similar strategies are applied widely in vitamin D genetic research.^24^

## Materials and methods

The Improving physical health and reducing substance use in psychosis (IMPACT)-randomized controlled trial is a clinical trial designed to evaluate the efficacy of a health intervention program in reducing the burden of heart disease, diabetes and stroke in patients with psychosis. The study is on the International Standard Randomized Controlled Trial Number (ISRCTN) online public registry (trial number: ISRCTN58667926; http://www.controlled-trials.com/ISRCTN58667926/IMPACT+RCT).

### Recruitment

Recruited patients met the following inclusion criteria: 18–65 years old and an ICD-10 diagnosis of psychotic disorder (F20–29, F31.2, F31.5). Overall, 224 ethnically-representative subjects within The South London and Maudsley NHS Trust gave informed consent before their participation in the study. The exclusion criteria were as follows: a primary diagnosis of learning disability, a co-existing physical health problem that would, in the opinion of the medical investigators, independently have an impact on metabolic measures and/or substance use habits, current pregnancy, mothers less than 6 months post partum and life-threatening or terminal medical conditions where intensive care is already provided.

### Diagnoses

Diagnoses were based on ICD-10 diagnostic criteria and were extracted from the documented diagnosis made by the treating consultant psychiatrist in the clinical notes at the time of recruitment.

### Blood extraction and biological assays

Consented patients provided blood samples for DNA analysis and metabolic profiling. DNA was extracted using the phenol–chloroform method. Genotypic status at rs703842 was determined using a custom-designed Taqman assay. Design of the assay probes used an 800-base pair region downloaded from the ENSEMBL website (http://www.ensembl.org). The sequence incorporated the regions flanking the rs703842 locus. The sequence was subsequently uploaded to the assay design feature in the Applied Biosystems website (https://www5.appliedbiosystems.com/tools/cadt/). Reaction products were run on a 7900HT sequence detection system (Applied Biosystems, Paisley, UK).

All 224 subjects yielded unambiguous genotyping results (100% call rate). The distribution of genotypes at rs703842 was in Hardy–Weinberg equilibrium (*P*=0.87). G (minor) allele frequency is consistent (0.33) across YRI and CEU Hapmap populations. Vitamin D levels (serum 25(OH)D) were determined with a chemiluminescence immunoassay (DiaSorin, S.P.A. Saluggia, Vercelli, Italy).

### Vitamin D status

In the analyses that follow, individuals with 25(OH)D levels below 10 ng/ml (<25 nmol/l) were classified as vitamin D deficient. Individuals above this threshold were considered ‘non-deficient’. This stringent interpretation of the literature^27–29^ takes account of the fact that the median vitamin D levels in schizophrenia are much lower than those found in the general population. For example, our own analyses reveal that the median vitamin D level in the 'non-deficiency' subgroup only reaches 16.6 ng/ml (see Results; Table 1). Thus, a threshold of 10 ng/ml^29^ optimizes the distribution of cases between comparison groups. Moreover, the 10-ng/ml threshold is physiologically relevant, as calcium absorption is known to decline rapidly below this serum concentration.^30,31^

### Covariates

Information on the following factors was collected: age at sampling, season of sampling, gender and self-reported ethnicity. Body mass index (BMI) was calculated using height and weight data collected at the time of recruitment. Total scores were calculated for International Physical Activity Questionnaire^32^ and Montgomery Asberg Depression Rating Scale.^33^ Information on medication (chlorpromazine equivalence) was also collected. Conversion of antipsychotic medication dose to chlorpromazine equivalence values was performed according to the established protocols.^34,35^ Percentage of the maximum daily chlorpromazine equivalent dose (the maximum daily dose of chlorpromazine) is equivalent to 1000 mg daily, as defined by the British National Formulary-licenced maximum dose.^36^

### Statistical analyses

Power calculations were performed using the software QUANTO^37^ to determine the numerical adequacy of the available sample (*n*=224) for the planned analyses, given expected genetic odds ratios in the range 1.7–2.0. On the basis of a log-additive genetic model, a prevalence of vitamin D deficiency of 65% in the schizophrenia population^38^ and a two-sided alpha of 0.05, statistical power was determined to be between 73 and 91%.

All other analyses were performed in STATA 12 (StataCorp, College Station, TX, USA) with two-tailed *P*-values reported. The Shapiro–Wilks test was used to assess normality of variables included in the analysis. The relationship between environmental factors and vitamin D status was then defined using the appropriate bivariate test (Independent samples *t*-test or Mann–Whitney). Environmental factors found to be discriminating of vitamin D status were incorporated into a final logistic regression model that included vitamin D status (deficiency: coded as ‘0’ or non-deficiency: coded as ‘1’) as outcome and age, gender and self-reported ethnicity. Ethnicity in these analyses was represented either as individual dummy variables (Caucasian, black/white mixed and African heritage), or as a single variable (using all white Caucasians as the reference group).

Raw genotypic values were recoded to reflect 0, 1 or 2 rs703842-*A* risk alleles (for MS). Logistic regression models were fitted to test for the effect of rs703842 genotype on the vitamin D status after adjustment for sociodemographic effects and environmental confounders.

### Ethics committee approval

Ethical approval for this study was obtained from The Joint South London and Maudsley and The Institute of Psychiatry NHS Research Ethics Committee. Ethical approval was granted on 17 July 2009 (REC Ref no. 09/H080/41).

## Results

A diagnostic breakdown of the cohort is provided in Table 1. Two-thirds of the total sample have an ICD-10 diagnosis of schizophrenia. The bulk of the remaining cases are split between schizoaffective and bipolar affective disorders. The combined number of delusional disorder and depression cases does not reach a double-digit proportion of the total sample (Table 1). We do not find evidence that the diagnostic composition of the cohort varies with respect to the vitamin D status, and this is true regardless of whether the diagnosis are kept split or grouped according to affective/non-affective status (*P⩾*0.15)

Table 2 provides a combined demographic, environmental and genetic overview of the study cohort. The ratio of males to females in the analyzed cohort (*n*=224) is almost 2:1. White British Caucasians represent the largest ethnic group, followed by individuals of African heritage. The remainder are of mixed White Caucasian/African heritage. The median age of the cohort is 45 (range in full sample: 23–66). As there were no significant differences in age or gender, the deficient and non-deficient groups are reasonably balanced in terms of demography (Table 2). Apart from the anticipated differences in plasma levels of 25(OH)D between deficient and non-deficient groups, patients with clinical vitamin D deficiency also tended to have a higher BMI, and score higher on the depressive symptom scale, compared with those who were non-deficient (Table 2). The direction of these differences is conventional in the sense that increasing depression symptoms, BMI and African heritage all correlate negatively with vitamin D at European geographical latitudes. Although these covariates have small effects, their aggregate effect on the vitamin D status in the logistic model is substantial (likelihood ratio statistics: *χ*^2^=91.7 (2 degrees of freedom (df)) *P*=1.2×10^−20^). Differences between the comparison groups in terms of age, medication, season of sampling, gender and ethnic makeup were not statistically meaningful (Table 2).

Table 3 illustrates the relationship between the rs703842-A risk allele (MS) and non-deficiency. It can be seen that the strength of the coefficient varies as different covariates are added to the logistic model (column 2, Table 3). The lowest *P* value achieved by modeling covariates individually is *P*=0.09. The aggregate effect of including age, gender, ethnicity, BMI and depressive symptoms together is reflected in the full-adjusted model. The result is suggestive of an underlying genetic effect (*ß*_standardized_=0.51, *P*=0.04). The genetic effect is directionally consistent across all the models in Table 3. The G allele mediates the drop in 25(OH)D to clinical deficiency (i.e. <10 ng/µl), while the A allele is protective. The variance attributable to rs703842 (0.049) represents 62% of the total variance explained in the final model (Pseudo *R*^2^=0.080). See Supplementary Table S1 for full logistic regression data.

## Discussion

Our results suggest that, in addition to being influenced by lifestyle changes that may accompany the onset of psychosis, vitamin D status in psychosis may also reflect genetic variation at rs703842, a known risk factor for MS.

Detection of the effect at rs703842 critically relies on the removal of biases relating to ethnicity, BMI and symptoms of depression (likelihood ratio statistics for the combined terms in the logistic model: *χ*^2^=91.7 (2df) *P*=1.2×10^−20^). Our subsequent analyses revealed the genetic association to be resilient to the addition of the remaining (unchosen) covariates in Table 2, namely International Physical Activity Questionnaire (outdoor physical activity), medication use and season of blood draw. This is to say that the genetic *P* value remains significant at the *P*<0.05 level when these covariates are added, individually or in combination, to the logistic model. The genetic effect can therefore be said to be additionally free of these potential confounders. It is important to note that such adjustment for environmental confounding is routine in vitamin D genetic research, e.g., Ahn *et al.,*^39^ Hiraki *et al.*^40^ and Ahn *et al.,*^41^ but is rare in schizophrenia research. A recent meta-analysis of vitamin D studies in schizophrenia helps to highlight this pitfall for the field.^42^ For example, the research question posed here would be intractable in most other schizophrenia cohorts, including those whose underlying objective is to study vitamin D. The inclusion of depressive symptoms in our model is unprecedented in the context of vitamin D research but can be justified for two clinical reasons: (i) depressive symptoms in schizophrenia are common^43–45^ and (ii) the inverse relationship between depressive symptoms and vitamin D levels is robust.^46,47^ Our results suggest that depressive symptoms experienced in psychosis and MS^44,48,49^ may contribute to vitamin D deficits reported at the clinical stages of illness.^18^

Our results highlight a curious discord between the observed effects of the rs703842 locus in psychosis and in MS; the *G* allele is associated with increased vitamin D levels in MS, however our analysis, undertaken in the context of psychosis, attributes the same effect to the *A* allele. It is therefore possible that our results reflect biological complexity of the sort that led the HLA alleles DRB1*03:01 and DQB1*02:01 to *increase* risk of MS and, at the same time, *decrease* the risk of schizophrenia in one recent genome-wide study.^5^ A similar picture emerged in a recent genome-wide bivariate analysis of *schizophrenia* and *height*,^50^ where it was found that the directional concordance between SNPs jointly associated with both traits does not reflect the directional relationship anticipated from prior observational work.^51,52^

Independent validation of our findings would suggest that shared genetics may help to explain convergence between the environmental risk profiles of psychosis and MS, based on season, geography and migration.^8–14^ Sustaining progress in this niche area of psychosis research will inevitably involve using genetics to test the credentials of vitamin D as an underlying risk factor. It is not possible to explore this within the current study context, due to the absence of controls. The key objective of such studies will be to leverage as much of the available vitamin D genetic architecture as possible using a mendelian randomization framework.

The *P* value for the genetic association is marginal and the corresponding explained variance only small (*P*=0.04, Pseudo *R*^2^=0.049); therefore, the failure to find methodologically compatible data sets for validation purposes is a limitation. Another issue is the failure to model all physiological confounders relevant to the research question posed. In particular, three hormones, calcitonin, parathyroid hormone (PTH) and prolactin, are known to stimulate increased levels of the bioactive 1,25(OH)D3 molecule through the altered expression of the *CYP27B1* gene (known also as 1α(OH)ase).^53,54^ Drugs that affect the hypothalamic dopamine system and/or pituitary dopamine receptors can enhance prolactin levels. Antipsychotic drugs fit into this category and are associated with a 2–10-fold increase in prolactin levels.^55^ The question of whether calcitonin or parathyroid is regulating 1α(OH)ase gene expression at any given time-point depends on the physiological status of calcium. For example, hypocalcemia causes PTH to be elevated, and this initiates the renal synthesis of 1,24(OH)_2_. However, at normal calcium levels PTH fails to stimulate the expression of 1α(OH)ase^54^ and is substituted in this role by calcitonin. Thus, the calcium status could potentially also be taken into account in conjunction with the three hormones. One final consideration relates to the assumption in our analysis that depression symptoms lead to low vitamin D, for it is also possible that muscular fatigue (and other symptoms associated with extreme deficiency) could provoke the onset of depression symptoms. We undertook *post hoc* analysis to understand the extent to which the main finding of the study is sensitive to uncertainty about this issue. The final logistic model was rerun, this time excluding Montgomery Asberg Depression Rating Scale scores. We established that in the absence of depression symptoms the genetic association diminishes only slightly (odds ratio=1.62 (0.98–2.68) *P*=0.061; pseudo *R*^2^=0.053); clearly not by enough to undermine our original diagnosis of a suggestive association (see Results). Thus, our preliminary findings suggest that the MS risk locus rs703842 may explain some of the variability of vitamin D status in established psychosis. The nature of the association found is consistent with the view that genetic variants linked with the vitamin D status can become obfuscated by confounding environmental factors; however, the conclusions reached by this study will require further validation in independent data sets.

## Figures and Tables

**Table 1:** Diagnostic breakdown of the sample

*Diagnosis*	*Full sample* n*=218*^a^ *% (*n*)*	*<10 ng/ml* n*=101 % (*n*)*	*⩾10 ng/ml* n*=117 % (*n*)*	*Test statistic*^b^
SCZ	67.4 (147)	72.3 (73)	63.3 (74)	
SZAD	14.2 (31)	15.8 (16)	12.8 (15)	
BPAD	14.7 (32)	8.9 (9)	19.7 (23)	Fisher's exact test *P*=0.173
Dep	2.75 (6)	2.0 (2)	3.42 (4)	
DEL	0.92 (2)	1.0 (1)	0.9 (1)	
Affective psychoses (Dep, BPAD, SZAD)	31.7 (69)	26.7 (27)	35.9 (42)	Pearson *Χ*^2^=2.1 *P*=0.15
Non-affective psychoses (SCZ, DEL)	68.4 (149)	73.3 (74)	64.1 (75)	

Abbreviations: BPAD, bipolar affective disorder; DEL, delusional disorder; Dep, depression; SCZ, schizophrenia; SZAD, schizoaffective disorder.

a Available diagnoses.

b For the comparison of diagnostic composition between deficiency/non-deficiency groups (columns 3 and 4).

**Table 2:** Clinical vitamin D status versus covariates

*Variable*	*Full sample* n*=224, median*	*<10 ng/ml,* n*=105, median (range)*	*⩾10 ng/ml,* n*=119, median (range)*	*Test statistic*^a^
25(OH)D (ng/ml)	10.45	7.2 (4.0–9.9)	16.6 (10.4–88.8)	*z*=−14.97, *P*<0.0001
Age	45	45 (25–66)	46 (23–66)	*t*=−0.389, *P*=0.70
Depression score (MADRS)	9	10 (0–35)	8 (0–37)	*z*=1.81, *P*=0.07
Body mass index (kg/m^2^)	30	31 (18–51)	29 (21–51)	*z*=1.72, *P*=0.08
IPAQ (outdoor activity)^b^	346.5	297 (0–4158)	396 (0–4158)	*z*=−0.168, *P*=0.09
Chlorpromazine (%max)^c^	40	30 (0–180)	40 (0–360)	*z*=0.49, *P*=0.63
				
	*Count (%)*	*Count (%)*	*Count (%)*	Χ*^2^-test statistics*
Female gender	81 (36.2%)	43 (40.9%)	38 (31.9%)	*Χ*^2^=1.9, *P*=0.16, df=1
				
*Ethnicity*				
Caucasian (white British)	106 (47.3%)	44 (41.9%)	62 (52.1%)	*Χ*^2^=5.56, *P*=0.06, df=3
Mixed (Caucasian/African heritage)	49 (21.9%	30 (28.6%)	19 (16.0%)	
African heritage	24 (10.7%)	13 (12.4%)	11 (9.2%)	
Other white caucasian	45 (20.1%)	18 (17.1%)	27 (22.7%)	
Sampling: spring/summer	105 (47.1%)	47 (44.8%)	58 (49.2%)	*Χ*^2^=0.43, *P*=0.51, df=1
				
*rs703842 genotype*				
GG	24 (10.7%)	12 (11.4%)	12 (10.1%)	
AG	97 (43.3%)	49 (46.7%)	48 (40.3%)	*Χ*^2^=1.33, *P*=0.52, df=2
AA	103 (46.0%)	44 (41.9%)	59 (49.6%)	

Abbreviations: df, degrees of freedom; IPAQ, International Physical Activity Questionnaire; MADRS, Montgomery Asberg Depression Rating Scale; 25(OH)D, 25-hydroxyvitamin D.

a On the basis of vitamin D group comparisons (deficiency/non-deficiency), independent samples *t*-test or Mann–Whitney test as appropriate.

b Total metabolic minutes per week.

c % Of the maximum daily chlorpromazine equivalent dose.

**Table 3:** The effect of covariate control on the rs703842 regression coefficient

*Covariate model (based on* N*=224)*	*Logistic regression*	
	*Standardized coefficient (95% CI)*	*Genetic* P *value*
1. Null (unadjusted)	0.20 (−0.19–0.60)	0.31
2. Adj. age	0.20 (−0.19–0.60)	0.30
3. Adj. gender	0.20 (−0.21–0.58)	0.36
4. Adj. ethnicity	0.40 (−0.07–0.87)	0.09
5. Adj. BMI	0.27 (−0.13–0.68)	0.19
6. Adj. depression symptoms	0.21 (−0.18–0.61)	0.29
7. Full adjusted	0.51 (0.02–1.02)	0.04

Abbreviations: Adj, adjusted; BMI, body mass index; CI, confidence interval.

The coefficients in the second column are standardized and reflect the relationship between rs703842 genotype and vitamin D status, under different covariate models (models 1–7). Deficiency is coded as ‘0’ and non-deficiency coded as ‘1’. Row 1 represents a null model in which the genetic effect is unadjusted for other modifiers. Rows 2–6 evaluate models containing single covariates. The genetic model in row 7 is adjusted for all covariates simultaneously.
