# Supplementary material for: Does environmental confounding mask pleiotropic effects of a multiple sclerosis susceptibility variant on vitamin D in psychosis?
Source: NPJ Schizophr. 2015 Oct 28;1:15036–. doi: 10.1038/npjschz.2015.36 (PMC4849459; doi:10.1038/npjschz.2015.36)
Supplement: Supplementary Table S1 [file npjschz201536-s1.doc]

Table S1. Full data for the full logistic model *

| **Variable** | **Odds Ratio** | **Std Err** | **95%CI** | **Z statistic** | **P value** |
| --- | --- | --- | --- | --- | --- |
|
| **rs703842-A allelic dosage** | 1.67 | 0.44 | 1.00 - 2.79 | 1.97 | 0.04 |
| **Mixed heritage (Caucasian/African)** | 0.33 | 0.13 | 0.15 - 0.73 | -2.73 | 0.01 |
| **African heritage** | 0.45 | 0.24 | 0.16 - 1.29 | -1.49 | 0.14 |
| **Winter blood sampling** | 0.74 | 0.25 | 0.38 - 1.44 | -0.88 | 0.38 |
| **Depression scores** | 0.98 | 0.02 | 0.94 - 1.01 | -1.41 | 0.16 |
| **BMI** | 0.97 | 0.02 | 0.92 - 1.02 | -1.34 | 0.18 |
| **Age** | 1.01 | 0.02 | 0.98 - 1.05 | 0.63 | 0.53 |
| **gender** | 0.89 | 0.32 | 0.44 - 1.78 | -0.33 | 0.74 |
| **% of maximum chlorpromazine equivalent dose** | 1.00 | 2.03 | 1.00 1.01 | 1.05 | 0.30 |

*N*=224; Likelihood ratio Chi^2^: 18.98 (9 degrees of freedom); P value = 0.025; Pseudo R^2^ for the rs703842 effect (model 7, table 3) = 0.049

*model 7 in table 3
